# Supplementary figures and images for: Evaluation of the Mexican warning label nutrient profile on food products marketed in Mexico in 2016 and 2017: A cross-sectional analysis
Source: PLoS Med. 2022 Apr 20;19(4):e1003968. doi: 10.1371/journal.pmed.1003968 (PMC9067899; doi:10.1371/journal.pmed.1003968)

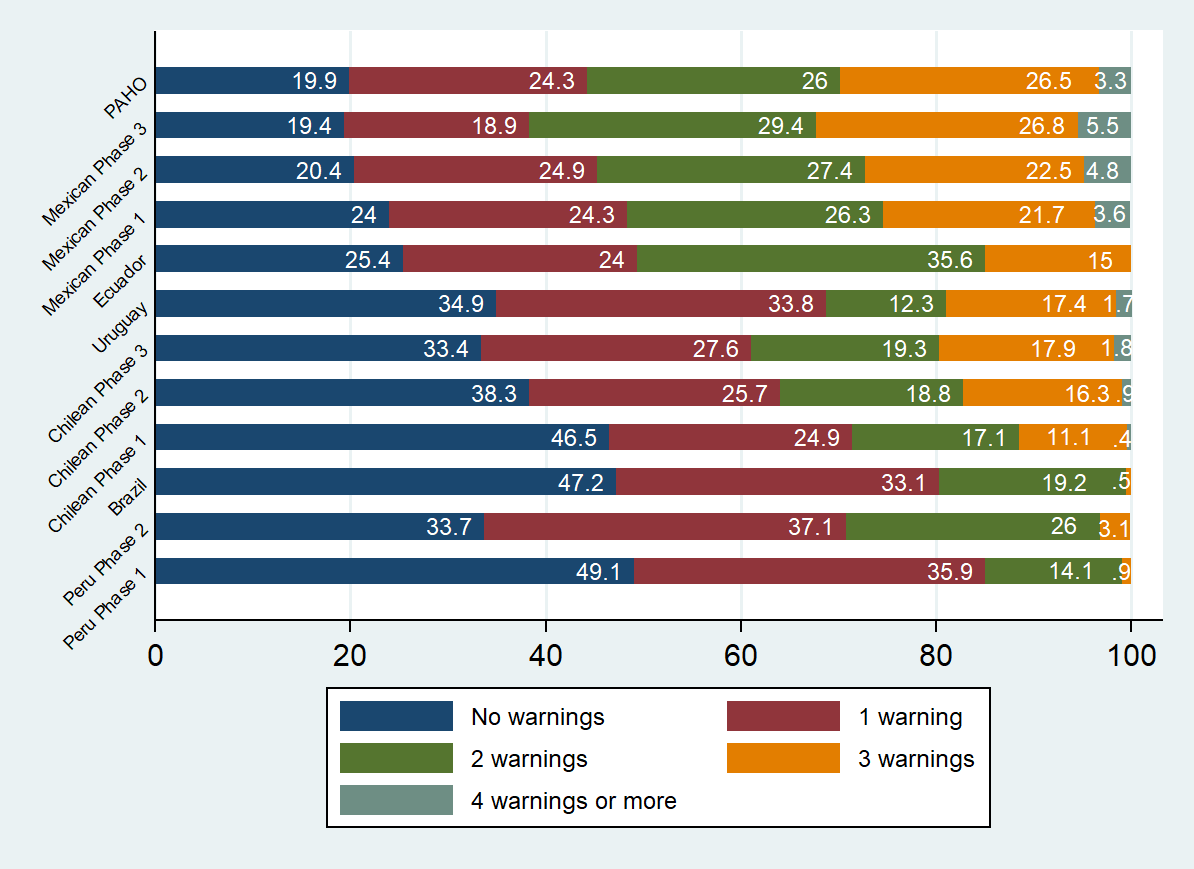

Supplement: S1 Fig — Overall number of warnings includes warnings in captions and warnings in octagons for the Mexican (0 to 7 warnings), Uruguay (0 to 4 warnings), Chilean (0 to 4 warnings), and Peru (0 to 3 warnings) nutrient profile models, and yellow and red traffic lights for Ecuador’s nutrient profile model (0 to 3 warnings). PAHO, Pan American Health Organization. *All comparisons were statistically significant (p < 0.05). (TIF) [file pmed.1003968.s002.tif]
