# Supplementary material for: Evaluation of the Mexican warning label nutrient profile on food products marketed in Mexico in 2016 and 2017: A cross-sectional analysis
Source: PLoS Med. 2022 Apr 20;19(4):e1003968. doi: 10.1371/journal.pmed.1003968 (PMC9067899; doi:10.1371/journal.pmed.1003968)
Supplement: S1 Table — *The sample comprises unique products. (DOCX) [file pmed.1003968.s003.docx]

| **Food group** | **Packaged foods included** | **N sample** | **%** |
| --- | --- | --- | --- |
| Eggs | Eggs, packaged egg whites | 41 | 0.11 |
| Cereals/Grains | Rice, quinoa, corn, oats, amaranth, flour, box cereal, granola, cereal bars (added with sugar), prepared cereals (e.g. paella), products derived from corn (e.g. tortillas, tostadas and tortilla chips) | 2,621 | 7.11 |
| Bakery | Prepared flour (added with sugar, fat and sodium), muffins, bread, cakes and pancakes | 464 | 1.26 |
| Fats/Oils | Oil, butter and margarine | 1,090 | 2.96 |
| Nuts/Seeds | Walnuts, peanuts, almonds and variations (with salt, natural, with chili, etc.) | 969 | 2.63 |
| Sugar/Sweets | Sugar, syrups, non-sugar sweeteners, flavorings (e.g. vanilla) | 521 | 1.41 |
| Condiments | Sauces, meat seasonings, herbs, salt-based seasonings (salt, salt and pepper, herb salt), garlic powder, chicken broth powder | 2,954 | 8.02 |
| Vegetables | Fresh vegetables (wheat sprouts, chopped vegetables, salads, celery sticks, etc.) and canned vegetables | 1,797 | 4.88 |
| Meat | Beef, pork, lamb, prepared meat (e.g. marinade or for hamburgers), bacon, sausages and ham | 944 | 2.70 |
| Sea Products | Packaged fresh seafood, canned tuna, sardines and salmon, preserved fish with salt | 1,141 | 3.10 |
| Soups | Pasta, prepared pasta soups, creams (e.g. mushrooms, corn, etc.), noodles and macaroni and cheese | 1,490 | 4.04 |
| Dairy Products | Cheeses, dairy ferments, yogurt and cream | 3,103 | 8.42 |
| Desserts/Toppings | Jams and jellies, flavored syrups (maple type), peanut butter and hazelnut cream | 958 | 2.60 |
| Combination Dishes | ready-to-eat and frozen (e.g. tacos, pizza, hamburgers, Mexican food, hot dogs, etc.). | 719 | 1.95 |
| Potatoes/Yams | French fries, mashed potatoes, and potato chips | 152 | 0.41 |
| Desserts | Sweets, jelly beans, chocolates, desserts (jellies and ice cream), caramelized fruit, cupcakes and cookies | 8,576 | 23.3 |
| Fruit/Juices | Packaged fruit, juices, canned fruit, and fruit-based snacks | 638 | 1.73 |
| Dairy Beverages | Plain milk and flavored milk | 450 | 1.22 |
| Non-Dairy Beverages | soft drinks, nectars, energy drinks, bottled tea, powdered drinks, water, mineral water, flavored drinks | 4,425 | 12.01 |
| Miscellaneous Items | Tea (bagged), coffee, herbal teas, cereal purees, cocoa and chocolate powder | 742 | 2.01 |
| Legumes | Beans | 452 | 1.23 |
| Snacks | Popcorn, Salted Cornmeal Fries, and French Fries | 2,026 | 5.50 |
| Poultry | Poultry, turkey ham and sausage, breasts, wings, chicken burgers (meat), nuggets | 521 | 1.41 |
| Overall | | 36,844 | 100 |
