# Supplementary material for: Evaluation of the Mexican warning label nutrient profile on food products marketed in Mexico in 2016 and 2017: A cross-sectional analysis
Source: PLoS Med. 2022 Apr 20;19(4):e1003968. doi: 10.1371/journal.pmed.1003968 (PMC9067899; doi:10.1371/journal.pmed.1003968)
Supplement: S2 Table — (DOCX) [file pmed.1003968.s004.docx]

| **Nutrient Profile Scheme/Latin American country** | **Aim** | **Food Categories** | **Foods Not Considered for Classification by the Nutrient Profile** | **Cut-Off Use** | **Ingredients of concern** | **Rationale/Basis * /Validation Method** |
| --- | --- | --- | --- | --- | --- | --- |
| Pan American Health Organization (PAHO) model [4]  Countries: Latin American Countries | Provide a tool to classify food and beverages with excess in free sugars, salt, total sugars, saturated fats, and trans fats to be used in the design and implementation of various regulatory strategies. | Processed and ultra-processed foods  No categories or food groups | Unprocessed or minimally processed foods.  Freshly prepared dishes, culinary ingredients (oils, sugar, honey, salt), breast milk substitutes, food supplements, alcoholic beverages | Threshold | (−): Sodium, free sugars, non-nutritive sweeteners, saturated fats, total fats, trans fats | Scientific-Based on WHO recommendations.  Validation of PAHO: Calibration [5]. |
| Multiple Traffic Light (Ecuador)[16]  Country: Ecuador | To provide clear and precise information about the content and characteristics of processed foods, without being misleading | All processed food for human consumption  No categories or food groups | Coffee, tea, aromatic herbs, vinegar, water, salt, alcoholic beverages.  Products whose natural content have fat, salt or sugars (with none of these nutrients added), formula and infant food, flours, food additives  Packaged produce (fruits, vegetables, chicken, meat, etc.) | Threshold  * The cut-off value is different if it is solid (gr) or liquid (ml.) | (−) Total fats, sugars, and salt. Legend for non-nutritive sweeteners | Regulatory-Based on Scientific evidence (PAHO, old criteria) in collaboration with industry.  Validation of Ecuador’s MTL: face [48, 49]. |
| Chilean Warning Octagons (2016, 2018 and 2019 criteria )[9,45]  Country: Chile | Provide clear and comprehensive information to the consumer on nutrients that, when consumed in excess, can cause health problems. | Apply to all national/imported packaged foods & beverages with added sodium, sugars, or saturated fat  No categories or food groups | Non-packaged foods and foods that do not have added sugars, saturated fats or sodium, products with a display panel area of 30cm^2^ | Threshold  * The cut-off value is different if it is solid (gr) or liquid (ml.) | (−) Energy, sodium, total sugar, saturated fat | Implement the thresholds progressively in a period of three years from most permissive (June, 2016) to current (June 2018) to future criteria similar to the PAHO model (June 2019).  Validation of CWO: face validity [46]. |
| Peruvian Warning label system.[12, 47]  Country: Peru | Provide simple and easy understanding information of nutrients of concern sodium. | Apply to all national/imported packaged foods & beverages with added sodium, sugars, or saturated fat  No categories or food groups | Non-packaged foods and foods that do not have added sugars, saturated fats or sodium, products with a display panel area of 50cm^2^ | Threshold  * The cut-off value is different if it is solid (gr) or liquid (ml.) | (−)Total sugar, saturated fat , trans fat, sodium. | Implement the thresholds progressively in 2 periods, 6 and 39 months after the approval.  Based on Chilean nutrient profile |
| Uruguayan Warning label System [13]  Country: Uruguay | Facilitate the identification of foods with high content of  nutrients associated with NCDs and promote healthier food choices. | Apply to all national/imported packaged foods & beverages with added sodium, sugars, or saturated fat  No categories or food groups | Non-packaged foods and foods that do not have added sugars, saturated fats or sodium | Threshold | (−)Sodium, saturated fat, total fat, total sugar. | Based on Chilean NP  Validation of PAHO profile flexibilization |
| Mexican Warning Label System [17]  Country: Mexico | Warn truthfully, clearly, quickly and simply about the content of critical nutrients that pose risks to your health in excessive consumption. | All processed and ultra-processed foods  No categories or food groups | Non-packaged foods and foods that do not have added sugars, saturated fats or sodium | Threshold | (−) Energy, sodium, saturated fat, trans fat, free sugars, added caffeine, non-sugar sweeteners. | Regulatory- Based on scientific and international recommendations, and tested through feasibility analysis. Based on PAHO model and Chilean nutrient profile [17]. |
| Brazilian Warning Label System proposal [50]  Country: Brazil | Inform simplified and standardized about the content of specific nutrients on the main panel of the food label, | Only apply to all national/imported packaged foods & beverages.  No categories or food groups | Non-packaged foods, non-processed foods, cheese, milk, frozen meat, fish or seafood, vinegar and vegetables oils; products with a display panel area of <40cm^2^ | Threshold* The cut-off value is different if it is solid (gr) or liquid (ml.) | (−) Added sugar, saturated fat, sodium. | Proposed by the National Health Surveillance Agency (ANVISA, for its acronym in Portuguese), based on WHO and Codex Alimentarius recommendations [50] |
