# Supplementary material for: Evaluation of the Mexican warning label nutrient profile on food products marketed in Mexico in 2016 and 2017: A cross-sectional analysis
Source: PLoS Med. 2022 Apr 20;19(4):e1003968. doi: 10.1371/journal.pmed.1003968 (PMC9067899; doi:10.1371/journal.pmed.1003968)
Supplement: S3 Table — All nutrient profiles were specified for 100 g or 100 mL. (DOCX) [file pmed.1003968.s005.docx]

|  | Eligibility | Energy | Sugar | Total fat | Saturated fat | Trans fat | Sodium | Other thresholds |
| --- | --- | --- | --- | --- | --- | --- | --- | --- |
| PAHO | Processed an ultra-processed foods and beverages | - | ≥10% of total energy from free sugars | ≥30% of total energy from total fat | ≥10% of total energy from saturated fat | ≥1% of total energy from trans fats | ≥1 mg of sodium per 1 kcal | Presence of  non-nutritive sweeteners |
| Mexican  Phase 1 | Foods and beverages with added free sugars (to evaluate energy and sugar), fat (to evaluate energy or fats) or sodium (to evaluate sodium) | ≥275 kcal/100 g; ≥70 kcal/100 ml or ≥10 kcal/100 ml from free sugars | ≥10% of total energy from free sugars except beverages with <10 kcal from free sugars | - | ≥10% of total energy from saturated fat | ≥1% of total energy from trans fats | ≥ 350 mg;  ≥45 mg for non-caloric beverages | Presence of  non-nutritive sweeteners and added caffeine |
| Mexican  Phase 2 |  | ≥275 kcal/100 g; ≥70 kcal/100 ml or ≥8 kcal/100 ml from free sugars | ≥10% of total energy from free sugars | - |  |  | ≥1 mg of sodium per 1 kcal or ≥300mg; ≥45mg for non-caloric beverages |  |
| Mexican  Phase 3 | Foods and beverages with added free sugars, fat or sodium |  |  |  |  |  |  |  |
| Chilean  Phase 1 | Foods and beverages with added free sugars (to evaluate energy and sugar), fat (to evaluate energy or fats) or sodium (to evaluate sodium) | ≥ 350 kcal/ 100 g; ≥100 /100 ml | Total sugar:  ≥22.5 g /100 g; ≥ 6g / 100 ml | - | ≥6 g / 100 g; ≥ 3 g / 100 ml | - | ≥800 mg /100 g; ≥100 mg /100 ml | - |
| Chilean  Phase 2 |  | ≥ 300 kcal /100 g; ≥80/ 100 ml | Total sugar:  ≥15 g /100 g; ≥ 5g 100 ml | - | ≥5 g/100 g; ≥ 3 g /100 ml | - | ≥500 mg /100 g; ≥100 mg/100 ml | - |
| Chilean  Phase 3 |  | ≥ 275 kcal /100 g; ≥70 kcal / 100 ml | Total sugar:  ≥10 g /100 g; ≥5g /100 ml | - | ≥4 g/100 g; ≥ 3 g /100 ml | - | ≥400 mg/100 g; ≥100 mg /100 ml | - |
| Ecuador | Foods and beverages with added free sugars, fat or sodium | - | Total sugar:  Low:  ≤5 g/100 g;  ≤2.5 g/100 ml  Medium:  >5 g to <15g/100 g;  >2.5 to <7.5g/100 ml  High:  ≥15 g/100 g;  ≥7.5 g/100 ml | Low:  ≤3 g/100 g;  ≤1.5 g/100 ml  Medium:  >3 g/100 g to <20 g/100 g;  >1.5 g/100 ml to <10 g/100 ml  High:  ≥20 g/100 g; ≥10 g/100 ml | - | - | Low  ≤120 mg /100  g or ml  Medium  >120 mg to <600 mg/100 g or ml  High:  ≥600 mg/100 g or ml | - |
| Peru  Phase1 | Processed foods | - | Total sugar:  ≥22.5 g/100 g; ≥6 g/100 ml | - | ≥6 g/100 g; ≥3 g/100 ml | Presence | ≥800 mg/100 ml; ≥100 g/100 ml | - |
| Peru  Phase 2 |  | - | Total sugar:  ≥10 g/100 g; ≥5 g/100 ml | - | ≥4 g/100 g; ≥3 g/100 ml | Presence | ≥400 mg/100 ml; ≥100 mg/100 ml | - |
| Uruguay | Foods and beverages with added free sugars (to evaluate sugar), fat (to evaluate fats) or sodium (to evaluate sodium) | - | Total sugar:  ≥10 g/100 g; 12 for foods with for foods ≤80% of energy from sugar and no added from non-sugar sweeteners.  3 g/ 100 ml; 5 g/ 100 ml for beverages with non-sugar sweeteners and 7g /100 ml for beverages ≤80% of energy from sugar and no added from non-sugar sweeteners | 9g / 100 g and 4 g / 100 ml | 4 g/ 100 gr and 3 gr/ 100 ml | - | 400 mg /100 g and 200 mg/ 100 ml | - |
| Brazil | All food and beverages except: fruits and vegetables, meats, salt, herbs, oil, sugar, coffee, baby food, or products with other nutrient restriction | - | ≥15 g/100 g from added sugars or ≥7.5 g/100 ml | - | ≥6 g/100 gr; ≥3 g/100 ml | - | ≥600 mg/100 g; ≥300 g/100 ml |  |
|  | | | | | | | | |
