# Supplementary material for: Evaluation of the Mexican warning label nutrient profile on food products marketed in Mexico in 2016 and 2017: A cross-sectional analysis
Source: PLoS Med. 2022 Apr 20;19(4):e1003968. doi: 10.1371/journal.pmed.1003968 (PMC9067899; doi:10.1371/journal.pmed.1003968)
Supplement: S4 Table — (DOCX) [file pmed.1003968.s006.docx]

| Ref. PAHO | All | Non-dairy beverages | Bakery | Combination dishes | Cereal/  grains | Potatoes/  Yams | Poultry | Marine products | Dairy product | Meat | Desserts | Sugar/  sweets | Legumes | Sauces/  condiments | Nuts/  seeds | Fats/  oils | Snacks | Toppings/  fillings | Vegetables | Soups | Miscellaneous items | Fruit/  juices | Dairy beverages |
| --- | --- | --- | --- | --- | --- | --- | --- | --- | --- | --- | --- | --- | --- | --- | --- | --- | --- | --- | --- | --- | --- | --- | --- |
|  |  |  |  |  |  |  |  |  |  |  |  |  |  |  |  |  |  |  |  |  |  |  |  |
| % Agreement | 57.4 | 31.9 | 32.1 | 37.5 | 41.3 | 43.8 | 44.4 | 44.6 | 54.6 | 52.5 | 56.1 | 61.5 | 60.9 | 63.8 | 67.8 | 77.3 | 74.1 | 74.5 | 76.5 | 81.9 | 91.1 | 95.8 | 69.2 |
| Kappa coefficient | 0.443 | 0.055 | 0.154 | 0.165 | 0.243 | 0.222 | 0.247 | 0.252 | 0.298 | 0.33 | 0.385 | 0.452 | 0.449 | 0.516 | 0.574 | 0.622 | 0.587 | 0.627 | 0.66 | 0.697 | 0.648 | 0.664 | 0.408 |
| Pearson correlation | 0.813 | 0.671 | 0.834 | 0.664 | 0.846 | 0.772 | 0.842 | 0.854 | 0.65 | 0.558 | 0.786 | 0.728 | 0.917 | 0.846 | 0.863 | 0.906 | 0.782 | 0.899 | 0.888 | 0.938 | 0.909 | 0.948 | 0.977 |
