# Supplementary material for: Evaluation of the Mexican warning label nutrient profile on food products marketed in Mexico in 2016 and 2017: A cross-sectional analysis
Source: PLoS Med. 2022 Apr 20;19(4):e1003968. doi: 10.1371/journal.pmed.1003968 (PMC9067899; doi:10.1371/journal.pmed.1003968)
Supplement: S5 Table — Pearson correlation coefficient (P) for number of warnings (Mexico, 0 to 7; Chile, Uruguay, and Peru, 0 to 4; Ecuador, 0 to 3; Brazil, 0 to 3; PAHO, 0 to 6): 0.00–0.30, negligible; 0.31–0.50, low; 0.51–0.70, moderate; 0.71–0.90, high; 0.91–1.00, very high (Adapted from: Hinkle D et al, 2003). All comparisons were statistically significant (p < 0.05). Percent agreement was assessed using kappa coefficients (k): 0%–100% healthy (without warnings) versus less healthy (1 or more warnings). Kappa coefficients were used to categorize agreement as follows: 0.01–0.20, slight; 0.21–0.40, fair; 0.41–0.60, moderate; 0.61–0.80, substantial; 0.81–0.99, near perfect (Viera A et al, 2005). (DOCX) [file pmed.1003968.s007.docx]

| n=37,902 | Mexican Phase 1 | Mexican Phase 2 | Mexican Phase 3 | Chilean Phase 1 | Chilean Phase 2 | Chilean Phase 3 | Ecuador | Peruvian Phase 1 | Peruvian Phase 2 | Brazilian | Uruguay |
| --- | --- | --- | --- | --- | --- | --- | --- | --- | --- | --- | --- |
| Mexican Phase 2 | P=0.963 k=0.893 96.3% | 1 |  |  |  |  |  |  |  |  |  |
| Mexican Phase 3 | P=0.906 k=0.861 95.3% | 0.941 k=0.957 98.9% | 1 |  |  |  |  |  |  |  |  |
| Chilean Phase 1 | P=0.739 k=0.515 76.6% | P=0.708 k=0.446 73.5% | P=0.643 k=0.425 72.5% | 1 |  |  |  |  |  |  |  |
| Chilean Phase 2 | 0.806 k=0.656 84.9% | P=0.756 k=0.576 81.8% | P=0.711 k=0.513 80.8% | P=0.916 k=0.830 91.6% | 1 |  |  |  |  |  |  |
| Chilean Phase 3 | P=0.818 k=0.754 89.8% | P=0.769 k=0.668 86.7% | P=0.733 k=0.648 85.8% | P=0.856 k=0.727  86.7% | P=0.923 k=0.892  95.0% | 1 |  |  |  |  |  |
| Ecuador | P=0.697 k=0.719  89.6% | P=0.692 k=0.764 91.7% | P=0.734  k=0.783 92.5% | P=0.635 k=0.542 77.9% | P=0.717 k=0.685 86.1% | P=0.745 k=0.784 91.0% | 1 |  |  |  |  |
| Peruvian  Phase 1 | P=0.636 k=0.409 70.7% | P=0.598 k=0.347 67.7% | P=0.622 k=0.340 67.4% | P=0.816 k=0.758  87.9% | P=0.741 k=0.658 82.9% | P=0.682 k=0.574 78.8% | P=0.549 k=0.457 73.1% | 1 |  |  |  |
| Peruvian  Phase 2 | P=0.727 k=0.636 84.9% | P=0.627 k=0.554  82.1% | P=0.715 k=0.548 82.2% | P=0.701 k=0.615 81.2% | P=0.797 k=0.757 88.3% | P=0.802 k=0.826 92.3% | P=0.736 k=0.709 87.8% | P=0.781 k=0.685 84.4% | 1 |  |  |
| Brazilian | P=0.654 k=0.459 73.8% | P=0.606 k=0.383  70.7% | P=0.655 k=0.383 70.3% | P=0.799 k=0.730 86.6% | P=0.839 k=0.749 87.6% | P=0.795 k=0.657 83.1% | P=0.930 k=0.508 76.1% | P=0.823 k=0.779  88.9% | P=0.815 k=0.708 85.7% | 1 |  |
| Uruguay | P=0.798 k=0.716 88.1% | P=0.754 k=0.634 85.1% | P=0.704 k=0.608  84.1% | P=0.818 k=0.707  85.7% | P=0.889 k=0.853 93.2% | P=0.914  k=0.922 96.5% | P=0.747 k=0.542 77.9% | P=0.674 k=0.582 79.2% | P=0.829 k=0.812 91.6% | P=0.780 k=0.678 84.2% | 1 |
| **PAHO** | **P= 0.715 k=0.765 91.9%** | **P=0.744 k=0.855 95.4%** | **P=0.813 k=0.861 95.6%** | **P=0.486 k=0.372 69.9%** | **P=0.552 k=0.479 57.2%** | **P=0.549 k=0.561 82.5%** | **P=0.665 k=0.707 89.8%** | **P=0.540 k=0.379 69.3%** | **P=0.676 k=0.604 59.9%** | **P=0.596 k=0.431 72.6%** | **P=0.643 k=0.571 82.6%** |
