# Supplementary material for: Evaluation of the Mexican warning label nutrient profile on food products marketed in Mexico in 2016 and 2017: A cross-sectional analysis
Source: PLoS Med. 2022 Apr 20;19(4):e1003968. doi: 10.1371/journal.pmed.1003968 (PMC9067899; doi:10.1371/journal.pmed.1003968)
Supplement: S1 Text — Codebook for photographic methods for measuring packaged food and beverage products in supermarkets and capturing information by fieldworkers. (DOCX) [file pmed.1003968.s008.docx]

# Codebook for Photographic Methods for Measuring Packaged Food and Beverage Products in Supermarkets and capturing information by field workers.

**Data collection**

After the selection of the observation sites by the criterion of population density in each of the main eight cities of the country, was developed the collection of photographs of the packages of food and beverage products from the more important commercial chains of supermarkets in Mexico; Bodega Aurrera, Chedraui, Costco, Mega Comercial Mexicana, Bodega Comercial Mexicana, Sams Club, Soriana, Superama, and Wal Mart Supercenter. These establishments selected had more than 100 employees and were within a population of more than 20,000 inhabitants within the buffer and a distance greater than 1,500 meters from the nearest establishment.

In each store selected, fieldworkers photograph all packaged food products available at the time. All categories and all brands, subsequently, duplicate products were removed. This strategy was followed to evaluate differences and availability of products by socio-economic status in which the store was located. The data collected by the fieldworkers included product information (e.g., company, brand), net content, price, nutrient facts panel information, ingredients list, health and nutrition claims, and FoPL, from photos of all sides of the packaging. Nutrition information was recorded and in the case of reconstituted products, the “as consumed” information was retrieved from the photographs of the products. The information contained in each photograph was captured using the software Research Electronic Data Capture (RedCap, Vanderbilt University, Tenn., US) and a field supervisor revised the completeness and accuracy of the captured data.

All packaged food and beverages were included in the photo collection. Taken together, 27 different food categories were identified initially: meat, sauces, fats/oils, beverages, salty snacks, sweet snacks, cereals/grains, fruit and vegetables, cheeses, other dairy products, bread, cookies, potatoes and pasta, corn made food, rice and other grains, legumes, flours, sea products, spreadable dressing, ready to eat foods, baby food, soups and creams, water, sweeteners, coffee, tea and, condiments.

For each food category, several subcategories were created depending on the large food group (from 1 category for cheeses to 14 categories for other dairy products). These food subcategories were re-categorized into the food and beverages groups (**S1 Table**) that allowed us to distinguish food products and beverages most likely subject to the food labelling.

Fieldworkers were instructed to take photographs of any packaged food and beverages products in their largest package size for products in which more than one package size was available. However, in the instances in which the food product used different marketing strategies depending on the package size, were took photos of all of sizes to capture the different marketing strategies (Kantar, et al). Photographs of food labels were taken from all sides of the food packages with the use of smart phones (Nokia, C1) during weekdays at the supermarkets. The photos included; bar code, package shape, volume or weight of the package, nutrition information table, and ingredients.

The quality control was performed at two levels, first by the fieldworkers in the moment of data collection in which they viewed the photo of the product in the smartphone and retake the photo if it was not clear enough. And after each day of data collection the field coordinator collected and review all of the photos for visibility and completeness making sure that the product’s photos included pictures with all sides of the product, the bar code, all of the ingredients, the Nutrition Fact label, the net content of the product as Kantar, et al. describe in their methodology. If some photos were not clear enough the fieldworkers had to re-take the selected low-quality photos the next day. All photos were stored on an external hard drive.

**Data management**

After the collection and store of the photographs, fieldworkers captured information using the software Research Electronic Data Capture (RedCap, Vanderbilt University, Tenn., US) and a field supervisor revised the completeness and accuracy of the captured data.

Important Considerations:

- Always use the “tab” key to enter any data inside a box.
- In any text box, always use CAPITAL LETTERS WITHOUT ACCENTS [for example: ñ and/or á; because the programs do not facilitate the use of these symbols, therefore everything written in the same format will greatly facilitate data analysis].
- Enter -888 in the boxes that request numbers if the data does not exist on the container.
- It is not necessary to enter all the data of a form at once, but the captured data should always be saved, even if you have not completed the entry of all the data requested within the form.

Steps to enter REDCap and navigate in the project:

1. Login to REDCap: <https://globalrc.ad.unc.edu/redcap/>
2. Go to “My projects” and enter the project: Mexico Bloomberg Pilot

**Data Capture**

Each product will have a unique ID.

- In case of having left the data entry incomplete in a previous session, these can be completed by entering them through the “Incomplete Records” menu. In this menu you can select the ID you want to finish capturing.

- To enter a new product you need to enter a new unique product ID into the box called “enter a new or existing unique product ID”. Then use the 'tab' key to enter this new ID.

- The next step will be to save this ID in the system. First, mark it as “Complete” and then click on “Save Record”

Double capture: barcode

1) Verify that you have entered REDCap using the user: Labeled1.

On this screen you must enter the barcode of the container and mark this form as "Complete". Then click on “Save Record”.

2) Then do a “log-out” of REDCap.

3) Login to REDCap using the user: Tagged2

On this screen you must enter the barcode of the container and mark this form as "Complete". Then click on “Save Record”.

4) Then log out of REDCap and re-login to REDCap using the username: Tagged1

5) Continue capturing the rest of the data for this product with the user: Tagged1

**PRODUCT PHOTOS**

In this section, we will explain the steps to upload to the server (cloud) some photos related to the product, whose data you will enter according to the following forms that come in the mask.

Please note for any photo (file) uploaded to REDCap, there is a file size limit of 1024 MB. This implies that any photo file must be prepared with a size that is suitable for REDCap before uploading it to the platform. Only one file can be uploaded per mailbox on REDCap.

“A photo of the front face”: You must upload the best photo you have of the front face of the product.

“A photo of the barcode”: You must upload the best photo you have of the product's barcode.

"Photo of the list of ingredients-1": You must upload the best photo you have of the list of ingredients of the product. If all the ingredients are not found in one photo, you must continue to the next REDCap box to upload more photos of the ingredients list.

"Photo of the list of ingredients-2": You must upload the best photo you have of the rest of the list of ingredients of the product. If not all ingredients are found in these two photos, you must upload the best two photos that have the highest proportion of the product's ingredient list.

**BASIC INFORMATION FORM**

“Photo Date”: You can enter the date of the photos that correspond to the container (product) you are entering.

“Food subcategory”: You can enter the code (an integer) of the subcategory to which the product you are capturing belongs. You must have a list of these subcategories with their codes, which have a range between 11 and 536.

“Container part of a multipack”: Select 1-yes or 0-no respectively, if the container is part of a multipack or not.

“Blurred photos”: Select yes or no, respectively, if one or some of the photos of the product for which advertising data is to be captured are blurred or if parts of the packaging cannot be read. For example, bright containers.

“Lost photos:”: Select yes or no, respectively, if it is clear that one or some of the photos of the product for which advertising data is to be entered are lost or prevent the entry of data for a part of the container. For example, when the nutritional table is not included, although they indicate that it exists.

“Supermarket code”: Capture the code of the supermarket where the photos of the product you are capturing were taken.

“Product Barcode Number”: Capture the product barcode (UPC) exactly as it is written on the package.

“Company of the product factory”: Capture the name of the company that has manufactured the product.

“The brand of the product”: Capture the brand of the product.

“Product name”: Capture the name of the product as it is written on the package, probably on the main face of the package.

“Observations”: In this box enter any comments or observations regarding this form and the requested data.

**GUIDELINE DAILY AMOUNTS**

“Presence nutritional seal”: If there is a nutritional seal on the container, mark 1-yes; if there is no nutritional seal on the container mark 0-no.

“Presence GDA-saturated fats”: If there is a GDA for saturated fats on the container, mark 1-yes; if there is no GDA for saturated fat on the container mark 0-no.

“Presence of GDA-other fats”: If there is a GDA of other fats in the container, mark 1-yes; if there is no GDA for other fats on the container mark 0-no.

“Presence GDA-total sugars”: If there is a GDA of total sugars in the container mark 1-yes; if there is no GDA for total sugars on the container, mark 0-no.

“Presence GDA-sodium”: If there is a sodium GDA in the container, mark 1-yes; if there is no sodium GDA on the container mark 0-no.

“Presence GDA-energy per container”: If there is a total energy GDA in the container, mark 1-yes; if there is no total energy GDA on the container mark 0-no.

“Presence GDA-energy per serving”: If there is a GDA of energy per serving per container, mark 1-yes; if there is no GDA for energy per serving per container mark 0-no.

**PORTIONS FORM**

“Container size”: Capture in numerical format the size of the total content that is on the container label (not the serving size). In the example it would be: 500.

“Pack size_unit”: Select the unit of the pack size (0-g or 1-mL).

“Size of portions indicated on the container (Number)”: In this box enter the size of the portion indicated on the container.

“Size of portions indicated on the container (Unit)”: In this box enter the unit that corresponds to the size of the portion indicated on the container, it must be in grams (g) or milliliters (mL). If it is in another unit that cannot be converted to these units, select the “other” option and then a box will appear to write the type of this unit.

“Number of servings per container”: In this box enter the number of servings indicated on the container. If there is no net content in the container, please enter -888 (do not leave the box blank).

*The warning message along with this red line comes because -888 is outside the valid range for a number of servings within the package.

The idea is that when a warning like this appears and it is NOT due to the entry of -888, notify the study coordinator to verify if this value (which causes this warning) is true or not.

“Requires product reconstitution”: 0-No or 1-Yes.

If so:

1) With reconstitution, how much does the product yield (Number): Capture the number (only) regarding the quantity that the package says would be the final product after reconstitution, or in other words it is called “the yield” (For example; 1/2 liter is 0.5 liters)

2) With reconstitution, how much powder of the product is required for the “yield” (Number): Enter the number (only) regarding the quantity that the package says that the powder is required to reach this “yield”. However, there will be cases where there are several portions of the "yield" within the same package (for example, a family package of whipped cream could contain 84 g to make two half liters of the product on two different occasions).

3) The instructions for the reconstitution of the product: write (in capital letters and without tildes or accents) the instructions for the reconstitution of the product that are written on the container.

A kind reminder: “always use CAPITAL LETTERS WITHOUT ACCENTS” [for example: ñ and/or á; because programs don't make it easy to use these symbols and everything written in the same format will make data analysis much easier].

“Observations”: In this box enter any comments or observations regarding this form and the requested data.

**INGREDIENTS SECTION**

During ingredient capture, when symbols such as "α" appear, you must capture it as the name "alpha".

“Declare list of ingredients”: 0-if there is no list of ingredients; and 1-if there is a list of ingredients on the product packaging.

“Characterizing ingredient 1-30”: There are boxes to enter up to 30 ingredients (in capital letters and without tildes or accents). NOTE: If there are more than 30 ingredients in the container, contact the project manager.

“Second list of ingredients”: SOME PRODUCTS CONTAIN INFORMATION ON PARTS OF THEIR INGREDIENTS. Select yes or no, whether or not there is a second list of ingredients on the container.

If yes:

1) “Name of the first list of ingredients”: Write in the box the name that belongs to the first list of ingredients (in this example it would be: YOGURT).

2) “Name of the second list of ingredients”: Write in the box the name that belongs to the second list of ingredients (in this example it would be: BLACKBERRY PREPARED).

3) “Characterizing ingredient 1-30”: There are boxes to enter up to 30 ingredients (in capital letters and without tildes or accents). If there are more than 30 ingredients in the container, contact the project manager.

“Third ingredient list”: Select yes or no, whether or not there is a third ingredient list on the container.

If yes:

1) “Name of the third list of ingredients”: Write in the box the name that belongs to the third list of ingredients.

2) “Characterizing ingredient 1-30”: There are boxes to enter up to 30 ingredients (in capital letters and without tildes or accents). If there are more than 30 ingredients in the container, contact the project manager.

“Observations”: In this box enter any comment or observation regarding this form and the requested data.

**NUTRITION FACTS**

“Nutrient declaration”: If the container contains information in any format regarding the amount of nutrients contained within the product, mark 1-Yes, such as: energy; fat (total; saturated; trans); cholesterol; sodium; Total carbohydrates; Dietary fiber; sugars; proteins. NOTE: After selecting Yes, you will see all of the following:

“How many nutrient declarations (NFPs):” Select the number of declarations (1 NFP, 2 NFPs, 3 NFPs, or more than 3 NFPs) of nutrients that exist on the package. For “normal” products that do not require reconstitution, they probably only have a single nutrient declaration (1 NFP). For products that require reconstitution, it is likely that they may have more than one nutrient declaration. For example, a soup powder containing one NFP from the package as purchased and a second NFP from the package as it would be prepared according to the reconstitution instructions.

Another example might be a powdered pudding that contains one NFP from the package as purchased and two more NFPs from the package as would be prepared according to directions for reconstitution with skim milk and/or whole milk.

If you select 2 NFPs or 3 NFPs or 'more than 3 NFPs' you will see a space to enter the second and/or third nutrient declaration that exists on the package.

“NFP # Nutrient Declarations Unit (NFP #)”: Select the unit that corresponds to the NFP you are entering: g, mL, or per serving.

“NFP (1-3)-As purchased or as it should be prepared:” In the previous Portions form, if you have registered that the product requires reconstitution, you will see the following boxes before the information of each nutritional table (NFP 1 -3) that will be captured for said product. That is, the form of the product referred to in the nutritional table whose data is shown in this box must be specified:

“Formato original” –Se refiere al producto en su formato original, como está en el envase al tiempo de la compra, sin ninguna reconstitución. Si selecciona ‘formato original’ debe seleccionar del menú que va a aparecer cuál es la recomendación de uso para reconstituir el producto. Si no está en el listado, seleccione la casilla ‘5-otro’ y después, va a ver otra casilla nueva NFP (1-3) ‘preparado con que-otro’ para escribir cuál es el producto que está recomendado para reconstituir el producto.

INSTEAD OF A COMMA (,)]. Enter the data as it is written exactly according to the column that corresponds to the amount per 100 g or 100 mL. If there is no information, enter -888 in the box.

“Saturated Fats [g in 100 g or 100 mL or per serving]”: [USING A PERIOD (.) INSTEAD OF A COMMA (,)]. Enter the data as it is written exactly according to the column that corresponds to the amount per 100 g or 100 mL. If there is no information, enter -888 in the box.

“Trans Fats [g in 100 g or 100 mL or per serving]”: [USING A PERIOD (.) INSTEAD OF A COMMA (,)]. Enter the data as it is written exactly according to the column that corresponds to the amount per 100 g or 100 mL. If there is no information, enter -888 in the box.

“Monounsaturated Fats [g in 100 g or 100 mL or per serving]”: [USING A PERIOD (.) INSTEAD OF A COMMA (,)]. Enter the data as it is written exactly according to the column that corresponds to the amount per 100 g or 100 mL. If there is no information, enter -888 in the box.

“Polyunsaturated Fats [g in 100 g or 100 mL or per serving]”: [USING A PERIOD (.) INSTEAD OF A COMMA (,)]. Enter the data as it is written exactly according to the column that corresponds to the amount per 100 g or 100 mL. If there is no information, enter -888 in the box.

“Total Carbohydrates [g in 100 g or 100 mL or per serving]”: [USING A PERIOD (.) INSTEAD OF A COMMA (,)]. Captures the data as it is written exactly according to the column that corresponds to the amount per 100 g or 100 mL. If there is no information, enter -888 in the box.

“Total sugars [g in 100 g or 100 mL or per serving]”: [USING A PERIOD (.) INSTEAD OF A COMMA (,)]. Enter the data as it is written exactly according to the column that corresponds to the amount per 100 g or 100 mL. If there is no information, enter -888 in the box.

“Added/added sugars [g in 100 g or 100 mL or per serving]”: [USING A PERIOD (.) INSTEAD OF A COMMA (,)]. Enter the data as it is written exactly according to the column that corresponds to the amount per 100 g or 100 mL. If there is no information, enter -888 in the box.

“Total dietary fiber [g in 100 g or 100 mL or per serving]”: [USING A PERIOD (.) INSTEAD OF A COMMA (,)]. Enter the data as it is written exactly according to the column that corresponds to the amount per 100 g or 100 mL. If there is no information, enter -888 in the box.

“Sodium [mg in 100 g or 100 mL or per serving]”: [USING A PERIOD (.) INSTEAD OF A COMMA (,)]. Enter the data as it is written exactly according to the column that corresponds to the amount per 100 g or 100 mL. If there is no information, enter -888 in the box.

“Salt [mg in 100 g or 100 mL or per serving]”: [USING A PERIOD (.) INSTEAD OF A COMMA (,)]. Enter the data as it is written exactly according to the column that corresponds to the amount per 100 g or 100 mL. If there is no information, enter -888 in the box.

“Calcium [mg in 100 g or 100 mL or per serving]”: [USING A PERIOD (.) INSTEAD OF A COMMA (,)]. Enter the data as it is written exactly according to the column that corresponds to the amount per 100 g or 100 mL. If there is no information, enter -888 in the box.

“Declare another less than or less than or equal to a nutrient-which:”: At the end of the boxes on the nutrients of a nutritional table (NFP). There are five boxes to check if any (up to five) nutrients are listed on a container's nutrition chart with a < (a less than) or ≤ (less than or equal) symbol. If so, you must enter the amount without the symbol in any of the nutrients mentioned above, and then, at the end, select from this menu which nutrient had the symbol of < (a less than) or ≤ (less than or equal) to the side of its value.

“Observations”: In this box enter any comment or observation regarding this form and the requested data.

**Price**

“Price (MXN)”: You can capture the price that corresponds to the photo of the container in MEXICAN PESOS.

**ADVERTISING SECTION**

“There is a message”: If there is any advertising message on the container, you must select 1-Yes; if there is no advertising message on the container, you should select 0-No. If there is at least one advertising message on the container, and therefore you selected 1-Yes, you will see the following box to enter up to 10 advertising messages that exist on the container.

1. “(1-10) Which message”: Write the text of the message in the box. A kind reminder to always use CAPITAL LETTERS WITHOUT ACCENTS [for example: ñ and/or á; because programs do not make it easy to use these symbols and everything written in the same format will make data analysis much easier].

*Form Status”: This is for the encoder; if you think you need to go back to the form to capture some more information, you can select “Incomplete”, otherwise you should select “Complete”.

Do not forget to save the partial or total capture of the boxes, click on the “Save Record” or “Save and Continue” buttons at the top or below the template.
