# Supplementary material for: Evaluation of the Mexican warning label nutrient profile on food products marketed in Mexico in 2016 and 2017: A cross-sectional analysis
Source: PLoS Med. 2022 Apr 20;19(4):e1003968. doi: 10.1371/journal.pmed.1003968 (PMC9067899; doi:10.1371/journal.pmed.1003968)
Supplement: Attachment — Submitted filename: 2021_Review for PLoS Medicine_MX Nutrient Profile.pdf [file pmed.1003968.s010.pdf]

Review for: *PLoS Medicine*

Title: Validation of the Mexican Warning Label Nutrient Profile

Manuscript number: PMEDICINE-D-21-02443_R1

June 28, 2021

TO THE AUTHORS:

Study Summary:

This paper aims to validate Mexico's new nutrient profile against PAHO standards and to compare it with other Latin American nutrient profiles. A major motivation for Mexico's new nutrient profile is to encourage the food and beverage industry to reformulate their products to conform to the nutrient standards in order to avoid the more punitive components of the law under which the nutrient profile was made (e.g., the front of package labels warning of the product's excessive sodium, etc…). A key argument for the present manuscript's project is the lack of clear nutrition standards, especially in the context of consumption of processed foods.

Overall Comments:

Overall, this study is well-done and has high potential impact on nutrition-related research and policymaking. There are a few critical conceptual and methodological details that should be provided about the study, as well as some sections that could use editing for clarity. These revisions should be strongly considered to improve confidence in the validity and transparency of the study.

Comments regarding the development of the nutrient profile:

Line 123: Please clarify what this means and how it precludes PAHO from setting a caloric threshold.

Lines 125-129: Use of the Chilean threshold for calories seem appropriate since PAHO does not have one, but a citation and a short sentence describing how Chile came up with their standard (that is problematic, as described in the next lines) should be added.

Lines 129-136: Confusing as written and missing citation. Specifically, I understood or was confused as follows:

- WHO sugar intake recommendation is <25 g/day
- Average beverage intake is 2,000 ml/day – [Whose average? Mexican adults? Is this only sugary beverages or all kinds including water? Clarify and add citation.]
- The above were combined to create a standardized rate of 1.25g per 100 ml, which was rounded up to 2g/100ml. – [Why round up? Several numbers later it seems that the goal was to get to a number that was close to 1 teaspoon or 5 grams, but why? And what is the justification for nearly doubling the rate in the formulation of the nutrient profile?]

Lines 137-150: Similarly confusing development of the standard for sodium. The manuscript claims that these are new guidelines, and yet earlier in the manuscript sodium was described as being part of the PAHO nutrient profile. Is the new Mexican addition the division of sodium limits for solid versus liquid? Or did PAHO update only the nutrient profile for sodium?

Line 140-142: Do you mean: "The mean value of added sodium was 45 mg per 100 ml"?

Line 144: Do you mean: "Consumption of the daily recommended allowance of 2 liters of water would mean healthy beverage consumption of 40 mg of sodium"?

- Excess sodium would be 45 mg per 100 ml for non-caloric beverages – Why isn't the nutrient profile limit 45 per 200 ml, which would be 5 mg over the allowance for pure water? Setting the limit at 45/100 is 2.5 times the water sodium – what is the guiding principle?

Comments about the mapping of retail products to nutrient profile

- Why did this project undertake human fieldwork and coding rather than looking at products sold? (minor comment)
- More details about the product selection process are needed.
    - How were retailers within each location selected? Centrally, or was it up to each fieldworker? What approach was used to ensure a common set of selections across sites?
    - Within each retailer, how did fieldworkers identify which products to photograph and code? What proportion of products overall and by site/retailer were selected and what was the justification for selecting the products?
- More details about the coding structure and process are needed.
    - The product codebook including photography instructions should be provided as an appendix.
    - Details about how fieldworkers were trained in product coding should be provided, including standards for ensuring inter-rater reliability.
    - What was the range of products included in the 38,872 products? Does that number represent a unique number of products? How did you know the products were unique?
- How confident are you about the accuracy of the food ingredient labeling on which this work is based? Some studies (apologies for not having the citations at hand) have suggested that the actual calorie count and nutrient content in packaged foods in the US vary rather substantially. If that is the case in Mexico, how would that affect the results of this study? I suspect variance would mean an overall bias in the food products to be perceived as healthier than they actually are, in which case your study would be even more conservative than it is in terms of identifying unhealthy foods. Still, this is worth considering in the discussion as a potential source of bias or limitation.

- Lines 355-363: This section (comparison of Durán study with present manuscript) is not clear.
    - If Durán study used a more stringent version of a nutrient profile, shouldn't their results find fewer healthy foods than the Mexico study reported in this manuscript?
    - Also, Durán study used Chile's final phase (i.e., the most stringent nutrient profile) compared with the present study using the Chile's Phase 1 nutrient profile.
        - What is the justification for comparing Mexico to Chile Phase 1 rather than Phase 3? If no compelling justification, analyses should be rerun with such comparison.
