# Supplementary material for: Evaluation of the Mexican warning label nutrient profile on food products marketed in Mexico in 2016 and 2017: A cross-sectional analysis
Source: PLoS Med. 2022 Apr 20;19(4):e1003968. doi: 10.1371/journal.pmed.1003968 (PMC9067899; doi:10.1371/journal.pmed.1003968)
Supplement: Attachment — Submitted filename: Response to Reviewers_Round1_final.docx [file pmed.1003968.s011.docx]

**Review for: PLoS Medicine**

Title: Validation of the Mexican Warning Label Nutrient Profile

Manuscript number: PMEDICINE-D-21-02443_R1

June 28, 2021

Due date to resubmit: March 1st, 2022.

# Requests from the editors:

Our academic editor commented: "I disagree with the term "validity" in this context. As the authors discuss, there is no gold standard to be considered ...", and we therefore suggest adapting the language used throughout the paper in favour of "evaluation" and "comparison" between models.

Answer: thank you for the comment. We have replaced the term “validity” for “evaluation” or “validate” to “evaluate”, or “was valid” to “was useful”.

Please adapt the title to better match journal style. We suggest: "Evaluation of the Mexican warning label nutrient profile: A population-based cohort study".

Answer: thank you for the suggestion. We don’t believe this is a cohort study because products were analyzed cross-sectionally. Therefore, we have modified the title as follows: Evaluation of the Mexican Warning Label Nutrient Profile: A cross-sectional analysis of the Mexican market share in 2016 and 2017.

Please restructure the abstract into three subsections (i.e., Background, Methods and findings, and Conclusions).

Answer: thank you for the comment. We have restructured the abstract as indicated.

Please add a new final sentence to the "Methods and findings" subsection, which should begin "Study limitations include ..." and should quote 2-3 of the study's main limitations.

Answer: thank you for the comment. We have added a sentence in the “methods and findings” subsection with the study’s main limitations.

“Study limitations include that warning legends (such as the addition of caffeine or non-caloric sweeteners) considered by the Mexican NP model were evaluated as if they were octagon warnings and our data is limited by the quality of the information reported in the list of ingredients and the nutrition facts table of the products.”

After the abstract, please add a new and accessible "Author summary" section in non-identical prose. You may find it helpful to consult one or two recent research papers in PLOS Medicine to get an impression of the preferred style.

Answer: thank you for the comment. We have included the “Author summary” section.

Author summary

Why was this study done?

• Nutrient profile models that reflect the nutritional quality of food products are needed to help governments and decision-makers seeking to adapt simplified front of package labeling systems in their countries.

• The calibration of a nutrient profile aims to compare whether the nutrient profile model classifies foods correctly against reference methods, increasing the evidence supporting the model and improving its confidence. The Pan American Health Organization (PAHO) has developed a model that may work as a reference for Latin American countries.

• Several nutrient profiles have been developed and implemented in Latin America, with scarce evidence about their performance using representative databases of products of the market share.

Recently, Mexico developed its own nutrient profile model based on the PAHO,and Chile Phase 3 models, nutritional scientific population-based recommendations and a feasibility analysis of products sold in the Mexican market share.

What did the researchers do and find?

•This study compared the classification of a sample of more than 38 thousand unique food products available in the Mexican market according to nutrient profile models from six countries (Mexico, Ecuador, Chile, Peru, Uruguay and Brazil) against the PAHO model.

• According to the PAHO model, the Mexican nutrient profile phase 3 performed best in identifying unhealthy products based on their content of energy, sugar, saturated fat, trans fat, sodium, addition of non-caloric sweeteners or caffeine. This model had a strong correlation with PAHO model (>91.9%).

• Our findings add support to the relevance of using the Mexican nutrient profile to evaluate the nutritional quality of food products as a basis for the prevention of obesity and chronic diseases related to diet.

Early in the Methods section (main text), please state whether or not the study had a protocol or prespecified analysis plan, and if so attach the relevant file, and referred to in the text.

Answer: thank you for the observation. We have included in the methods section information regarding the protocol of the study and the main project.

“The protocol of this study was approved by the Research, Ethics and Biosafety Committees of the Mexican National Institute of Public Health (approved number: 1530). This study was part of the study INFORMAS: Monitoring and benchmarking food environments globally [32].”

Please correct "strengths" at line 461.

Answer: thank you for the comment. We have corrected the word.

Please avoid assertions such as "the first" at line 464, and where needed add "to our knowledge" or similar.

Answer: thank you for the comment. We have included “to our knowledge” in the sentence.

“Also, to our knowledge this is the first study comparing an almost exhaustive number of NP models used in Latin America, filling the knowledge gap regarding the comparability ~~and validity~~ of these nutrient profiles.”

Throughout the text, please reformat reference call-outs as follows: "... and calories [1,2]." (noting the absence of spaces within the square brackets).

Answer: Thank you for the comment. We have reformatted reference call-outs.

Please remove all iterations of "[Internet]" from the reference list.

Answer: Thank you for the observation. We have removed iterations of “[Internet]” from the reference list.

Please revisit reference 6, which may be lacking a journal name or URL.

Answer: Thank you for the observation, the reference 6 is not missing of journal, but we have included the URL.

Please include a completed checklist for the most appropriate reporting guideline, e.g., STROBE, as an attached file, labelled "S1_STROBE_Checklist" or similar and referred to as such in the Methods section (main text). In the checklist, please refer to individual items by section (e.g., "Methods") and paragraph number, not by line or page numbers as these generally change in the event of publication.

Answer: thank you for the comment. We have included a file with the Strobe checklist. (S5 Strobe checklist)

Thank you for supplying the attached data file. However, this may be challenging to access, and we suggest providing Excel files instead, or using an online repository.

Answer: Our apologies, we did not understand which file we have to attach in excel format.

# Comments from the reviewers:

# *** Reviewer #1:

Review of Validation of the Mexican Warning Label Nutrient Profile.

The authors compare the agreement and correlation of the Mexican NP system with the Pan American recommendations and those of other Latin American countries using Cohen's kappa and Pearson's correlation. The sample size is large and impressive. The graphs are clear and make understanding of this complex issue easier to grasp. The article is over-all well-written and well-presented. I have one minor and one major recommendation.

Methods:

Line 174: Probably should be 'We selected a convenience sample'. Some indication as to how the stores were selected should be included, even if it's just "We chose stores within walking distance of the university" or whatever.

Answer: thank you for the comment. We have included in the method section a detailed description of the selection of supermarkets as well an appendix with the codebook of the photo collection.

“We ~~conveniently~~ selected a convenience sample of 127 and 136 retail stores in 2016 and 2017, respectively (from January 23, 2016 to December 15, 2017). Retail stores were geographically located in areas with high population densities. In the first place, the National Statistical Directory of Economic Units [35] was used to identify all economic units classified as "Retail trade in supermarkets" within each city. Of these, all hypermarkets, supermarkets, and convenience stores were initially selected. Then, a 1000-meter buffer around each establishment was drawn, and the total population within the buffer according to the 2010 census was calculated [36]., Subsequently, the distance to the nearest retail store establishment was calculated. All retail stores with a population density of > 20,000 inhabitants within the 1000-m buffer and with a distance of >1,500 meters from to the nearest retail store were included. Regardless of the above criteria, all membership food stores from the top grocery retailers in Mexico were included because these establishments market select brand products which are only available within these stores. The included food retailers altogether represent more than 70% of the market share in the country [37]. The final number of stores visited in each city varied, from 3 in Cuernavaca to 65 in Mexico City.

Within each store selected, fieldworkers photographed all packaged food products available at the time, including all food categories and all brands. ~~, subsequently, duplicate products were removed.~~ This strategy was followed because we were interested in to evaluating differences in the availability of products according to the socio-economic status of the neighborhood in which the store was located. , S~~s~~ubsequently, duplicate products were removed. The data collected by the fieldworkers included product information (e.g., company, brand), net content, price, nutrient facts panel information, ingredients list, health and nutrition claims, and FoPL, from photos of all sides of the packaging. Nutrition information was recorded and in case of reconstituted products, the “as consumed” information was retrieved from the photographs of the products. ~~The information contained in each photograph was captured using the software Research Electronic Data Capture (RedCap, Vanderbilt University, Tenn., US) and a~~ F~~f~~ield supervisor revised the completeness and accuracy of the captured data.”

Results:

No p-values or confidence intervals are reported anywhere in the article. This is somewhat understandable due to the density of the data presentation and the large sample size is likely to produce all significant p-values. However, this is a grave lapse in rigorous scientific reporting and it is essential that either p-values or confidence intervals are reported. STATA should produce both p-values and confidence intervals for both the Agreement and Correlation analyses. At a minimum, if all p-values are less than 0.001 (for example), then this should be stated somewhere in the text and in the table headings where kappa and the correlation are reported.

Answer: thank you for the comment. As the reviewer suggested, all comparisons were statistically significant. In supplementary table 4 is stated “All comparisons were statistically significant (p<0.05).” We have included this information in the tables and graphs.

# *** Reviewer #2:

Thank you for your comments and suggestions. The manuscript has improved significantly with your contributions.

Comments regarding the development of the nutrient profile:

- Line 123: Please clarify what this means and how it precludes PAHO from setting a caloric threshold.

Answer: Thank you for the comment, we have included the reason why the PAHO model does not have a threshold for calories.

“The PAHO model proposes thresholds for six critical nutrients (i.e, sugar, total fat, saturated fat, trans fat, sodium, and non-nutritive sweeteners), ~~but it does not have a threshold for calories since and, it is~~ based on ~~the proportional contribution to the energy of the product and on~~ the population nutrient intake goals for preventing diet-related chronic diseases. ; T~~t~~hese goals do not include a threshold for calories but are based on the energy contribution of each critical nutrient. In line with the former, the PAHO model considers specific thresholds for each critical nutrient based on their proportional contribution to the energy of the product, but it does not have a threshold for calories [16,20,21].”

- Lines 125-129: Use of the Chilean threshold for calories seem appropriate since PAHO does not have one, but a citation and a short sentence describing how Chile came up with their standard (that is problematic, as described in the next lines) should be added.

Answer: Thank you for the comment. We have included in the manuscript an explanation of the methodology used in Chile for establishing the ‘high in calories’ threshold and the citation.

“Hence, the Chilean NP model threshold for ‘high in calories’ was used for beverages and for solids in the Mexican NP model. The Chilean threshold for calories was determined according to the energy content in natural foods and beverages. For solids, the calorie threshold corresponds to the 90-95 percentile value of the energy distribution per 100 g of a natural food composition database [22]; this value has also been used to determine energy-dense foods [23]. For beverages, the Chilean model considers the energy content of plain milk per 100 mL (70 kcal) ~~was taken~~ as ~~a~~the reference [11,24]. Nevertheless, according to ~~limitation~~ of this threshold ~~is the fact that~~ some dairy beverages are classified as ‘High in calories’ and ‘High in sugars’, while soft drinks are only labeled as ‘High in sugars’, indicating that soft drinks are healthier than dairy beverages [25]. This is a controversial ~~situation~~ since all calories in soft drinks come from added sugar, while for dairy sweetened beverages other nutrients also account for total calories (e.g., lactose). Indeed, soft drinks have been directly linked with the development of some non-communicable diseases, like obesity and diabetes [21,26].”

- Lines 129-136: Confusing as written and missing citation. Specifically, I understood or was confused as follows:
- WHO sugar intake recommendation is <25 g/day.

Answer: Thank you for the comment and for highlighting this inconsistency. We have revised the manuscript to correctly reflect the scientific bases used for the development of our threshold. We have revised the document to reflect that the WHO recommendation to reduce daily intake of free sugars to less than 10% of total energy intake (equivalent to 50 g of sugar per day for an average diet of 2000 kcal) was used. See the new paragraph in the manuscript below.

- Average beverage intake is 2,000 ml/day – [Whose average? Mexican adults? Is this only sugary beverages or all kinds including water? Clarify and add citation.]

Answer: Thank you for the comment. We have revised this section and included additional information to clarify reviewer’s questions. See the new paragraph below.

- The above were combined to create a standardized rate of 1.25g per 100 ml, which was rounded up to 2g/100ml. – [Why round up? Several numbers later it seems that the goal was to get to a number that was close to 1 teaspoon or 5 grams, but why? And what is the justification for nearly doubling the rate in the formulation of the nutrient profile?]

Answer: Thank you for the comment and for highlighting this issue. We have revised this section and included additional information to clarify reviewer’s questions. See the new paragraph below.

“To solve this limitation, the Mexican NP model proposes a new cut-off point for the ‘Excess calories’ threshold in beverages. This criterion~~a~~ was developed considering two population scientific bases.; Tthe first one was the WHO recommendation to reduce daily intake of free sugars to less than 10% of total energy intake (equivalent to 50 g of sugar per day) [27,28]. The second scientific base was as well as the daily average beverage intake of 252~~00~~0 ml/day; this volume was established by the Beverage Consumption Recommendations for the Mexican Population, considering a diet that provides 2,200 calories and an adequate intake of all essential nutrients [29]. By dividing the WHO recommended daily intake of free sugars by the daily average beverage intake recommendation in Mexico, resulting in a standardized rate of 1.98~~25~~ g of sugar per 100 milliliters was obtained. For ease of calculations, t~~T~~his figure was rounded up into 2 grams/100 ml, equivalent to a <8 kcal per 100 milliliters (approximately 5 grams of sugar or one teaspoon in 250 ml), and set as ~~for~~ the final ~~second~~ ~~and third phases~~ thresholds of the ‘Excess of calories’ for the Mexican NP model. However, for the first implementation phase ~~;~~ of the Mexican FOPL, ~~for phase 1,~~ a limit of <10 kcal per 100 milliliters was established. This threshold was arbitrary and considered in order to provide manufacturers enough time to reformulate their products.”

- Lines 137-150: Similarly confusing development of the standard for sodium. The manuscript claims that these are new guidelines, and yet earlier in the manuscript sodium was described as being part of the PAHO nutrient profile. Is the new Mexican addition the division of sodium limits for solid versus liquid? Or did PAHO update only the nutrient profile for sodium?

Answer: thank you for your comment. We have rephrased the explanation for the development of the sodium criteria to clearly state that by the time when the threshold for non-caloric beverages was developed, the updated PAHO thresholds for sodium for solid and liquid products had not been released yet. Later, when PAHO released the new recommendation, the threshold for solids and liquids was used for the Mexican NP model, but we used our developed threshold for non-caloric beverages since it was very similar to the one proposed by PAHO for these beverages.

“Besides, ~~a new~~ thresholds for excess of sodium in solid and liquid products ~~was~~ were established~~. in two, B~~based on a ~~recent~~available PAHO ~~document~~recommendations at the time [4], a threshold of >1mg of sodium per 1 kilocalorie was initially established for solid and liquid products [30]. However, the proposed threshold had the limitation that it classified some low-calorie beverages with minimum amounts of added sodium (e.g., diet soda) as ‘High in sodium’. For example, a low-calorie beverage of 5 kcal and 10 mg of sodium would not comply with this threshold. Therefore, we developed a new threshold for non-caloric beverages based on the maximum. The content of added sodium in beverages available in the Mexican market. First, all beverages not exceeding the sodium criteria (>1mg/kcal) proposed by PAHO were selected (n=425, 85% were carbonated beverages). Then, we analyzed the sodium content per 100 mL of these beverages finding that the maximum content was of 45 mg [26]. ~~This is approximately twice the sodium content occurring naturally in drinking-water, which contains 20 mg of sodium per liter [31].~~

~~This threshold would allow a more stringent identification of products with excess of sodium, especially desserts, snacks, and sauces/condiments. A sample of 425 regular carbonated beverages complying with the PAHO threshold for sodium was analyzed, obtaining a value of 45 mg in 100 ml. Considering the recommended water daily consumption of 2 liters[29], this would lead to an intake of about a 40 mg of sodium per day [32].,~~ We established ~~Thus~~, a threshold of 45 mg/100ml of sodium ~~was used~~ as the criteria for the ‘Excess sodium’ label for non-caloric beverages. This threshold is similar to the 2020 PAHO threshold for sodium for ultra-processed and processed drinks that provide no energy (40 mg/100 ml) which was published later on [30]. Based on the updated PAHO thresholds, we decided to additionally consider the 300 mg of sodium in 100 grams/milliliters as the sodium threshold for solid and liquid product [30]. For the rest of food products, a threshold of 300 mg of sodium in 100 grams or milliliters was proposed, based on PAHO recommendations for the design of NP.25 This threshold would allow a more stringent identification of products with excess of sodium, especially desserts, snacks, and sauces/condiments. ~~.Finally, in accordance to the Mexican FoPL regulation, which considers a three-phase implementation process (2020, 2023 and 2025), three NP were proposed, with stricter nutrient criteria for each progressive phase.8.16 –~~ For the first phase of implementation a more permissive threshold of 350 mg per 100 grams or milliliters was established, and subsequent phases considered the proposed more stringent criteria.

- Line 140-142: Do you mean: “The mean value of added sodium was 45 mg per 100 ml”?

Answer: Thank you for the observation. We corrected the sentence to state that the value of 45 mg of sodium is not a mean, but a maximum content in the sample; also, this does not consider added sodium, but total sodium content.

- Line 144: Do you mean: “Consumption of the daily recommended allowance of 2 liters of water would mean healthy beverage consumption of 40 mg of sodium”?

Answer: Thank you for the suggestion, and for highlighting this issue. We have deleted the sentence since our threshold for sodium in non-caloric beverages is not based on sodium intake from water.

- Excess sodium would be 45 mg per 100 ml for non-caloric beverages – Why isn’t the nutrient profile limit 45 per 200 ml, which would be 5 mg over the allowance for pure water? Setting the limit at 45/100 is 2.5 times the water sodium – what is the guiding principle?

Answer: Thank you for the suggestion. We have now edited this paragraph to describe how this threshold was developed. The sodium content in pure water was not considered as a reference, but for comparative purposes. However, we now realize this may be confusing for the reader. We have now deleted all sections in this paragraph referring to sodium content in water and reinforced that this threshold is aligned with PAHO updated recommendations.

Comments about the mapping of retail products to nutrient profile:

- Why did this project undertake human fieldwork and coding rather than looking at products sold? (minor comment)

Answer: In Mexico, the government databases of products sold (INEGI) do not have complete or detailed information about the nutritional content and do not provide an estimation of retail data. Other private databases like Kantar or Nielsen are based on urban household purchases and do not include nutritional composition information. Hence, we collected information on the nutritional composition of a representative sample of products available in the Mexican market.

“Public databases containing information on nutrient composition and ingredient information on branded foods and beverages in Mexico are not available at the moment of the study. Therefore, nutrient composition ~~D~~data on~~f~~ the nutrition content of 38,872 packaged food products retailed in the Mexican market were collected from 2016 to 2017 following standardized procedures for measuring packaged foods and beverages according to Kanter et al, consisting of capturing selected product photographs at the points of sale, followed by information download and data entry at the office The information contained in each photograph was captured using the software Research Electronic Data Capture (RedCap, Vanderbilt University, Tenn., US) (Appendix 1) [31].”

- More details about the product selection process are needed.

- How were retailers within each location selected? Centrally, or was it up to each fieldworker? What approach was used to ensure a common set of selections across sites?

Answer: Thank you for the observation, we have added detailed information about the selection of retailers based on objective measures of population density.

“We ~~conveniently~~ selected a convenience sample of 127 and 136 retail stores in 2016 and 2017, respectively (from January 23, 2016 to December 15, 2017). Retail stores were geographically located in areas with high population densities. In the first place, the National Statistical Directory of Economic Units [35] was used to identify all economic units classified as "Retail trade in supermarkets" within each city. Of these, all hypermarkets, supermarkets, and convenience stores were initially selected. Then, a 1000-meter buffer around each establishment was drawn, and the total population within the buffer according to the 2010 census was calculated [36]., Subsequently, the distance to the nearest retail store establishment was calculated. All retail stores with a population density of > 20,000 inhabitants within the 1000-m buffer and with a distance of >1,500 meters from to the nearest retail store were included. Regardless of the above criteria, all membership food stores from the top grocery retailers in Mexico were included because these establishments market select brand products which are only available within these stores. The included food retailers altogether represent more than 70% of the market share in the country [37]. The final number of stores visited in each city varied, from 3 in Cuernavaca to 65 in Mexico City.”

- Within each retailer, how did fieldworkers identify which products to photograph and code? What proportion of products overall and by site/retailer were selected and what was the justification for selecting the products?

Answer: Thank you for the question. Since a secondary objective of this data collection process was to evaluate differences in the availability of products according to the socio-economic status of the neighborhood in which the store was located, fieldworkers were instructed to collect all products available at the aisles of the store. We have included this information in the manuscript.

In each store selected, fieldworkers photograph all packaged food products available at the time. All categories and all brands, subsequently, duplicate products were removed. This strategy was followed to evaluate differences and availability of products by socio-economic status in which the store was located.

“Within each store selected, fieldworkers photographed all packaged food products available at the time, including all food categories and all brands. ~~, subsequently, duplicate products were removed.~~ This strategy was followed because we were interested in to evaluating differences in the availability of products according to the socio-economic status of the neighborhood in which the store was located. , S~~s~~ubsequently, duplicate products were removed. The data collected by the fieldworkers included product information (e.g., company, brand), net content, price, nutrient facts panel information, ingredients list, health and nutrition claims, and FoPL, from photos of all sides of the packaging. Nutrition information was recorded and in case of reconstituted products, the “as consumed” information was retrieved from the photographs of the products. ~~The information contained in each photograph was captured using the software Research Electronic Data Capture (RedCap, Vanderbilt University, Tenn., US) and a~~ F~~f~~ield supervisor revised the completeness and accuracy of the captured data.”

- More details about the coding structure and process are needed.

- The product codebook including photography instructions should be provided as an appendix.

Answer: We have included an appendix with the codebook for fieldworkers.

- Details about how fieldworkers were trained in product coding should be provided, including standards for ensuring inter-rater reliability.

Answer: We have added information regarding fieldworkers training.

“Fieldworkers were trained nutritionists who attended a one-month workshop [33], which consisted in training and experiential learning on food composition, food labeling, and food promotion of packaged foods. In addition, fieldworkers received a one-day photography workshop led by a professional photographer, and half-week ~~a day~~ field-work training in a real supermarket. To standardize data collection processes, fieldworkers were provided with 50 products from different categories and varying shapes and sizes. Products were placed on a table in a large classroom and field workers were instructed to photograph the products, download images in their computers and capture data using RedCap [34]. Data captures were compared and filed-workers were considered as trained when inter-rater reliability was >80%.”

- What was the range of products included in the 38,872 products? Does that number represent a unique number of products? How did you know the products were unique?

Answer: Thank you for the observation, yes the 38,872 products were the unique number of products. The database was cleaned and processed using barcodes, name and brand of each product in order to ensure only information from unique products was considered. In supplementary table 1 we have included information of the sample size and percentage of the sample by food groups. In total, we included 36,838 unique products with complete nutritional information. A footnote was included to clarify that this number represents unique products.

- How confident are you about the accuracy of the food ingredient labeling on which this work is based? Some studies (apologies for not having the citations at hand) have suggested that the actual calorie count and nutrient content in packaged foods in the US vary rather substantially. If that is the case in Mexico, how would that affect the results of this study? I suspect variance would mean an overall bias in the food products to be perceived as healthier than they actually are, in which case your study would be even more conservative than it is in terms of identifying unhealthy foods. Still, this is worth considering in the discussion as a potential source of bias or limitation.

Answer: Thanks for the observation. The Mexican food labeling regulation during the data collection period (2016-2017) included rounding specifications for energy and nutrient reporting when these values were relatively low (e.g. manufacturers could report 0 g of sugar when the content was less or equal to 0.5 g per serving). This represents a limitation in our study because the number of products classified as less healthy could be higher. We add some lines in the limitations section:

“Second, our data is limited by the quality of the information reported in the list of ingredients and the nutrition facts table of the products. The regulation in force during the data collection period allowed zero values to be reported for energy and nutrients when their content was very small (i.e., small products such as candies reported servings of 1 g of sugar per portion). This was more common in products with small portion sizes, opening the possibility for underreporting. Nevertheless, the Federal Commission for the Protection against Sanitary Risks (COFEPRIS by its acronym in Spanish) in Mexico monitors and assesses the nutrition composition and labelling of packaged foods and beverages, this preventing manufacturers to report incorrect information on the labels. Additionally, food manufacturers are required to submit a bromatology analysis of their product before it reaches the Mexican market. Due to that process, we reckon that the content declared is accurate in general. However, no national assessment about the accuracy of packaged foods has been published. As with all food composition databases there is a risk of error. Under this assumption, our results are conservative, and the number of foods and beverages classified as less healthy could be higher.”

- Lines 355-363: This section (comparison of Durán study with present manuscript) is not clear.

If Durán study used a more stringent version of a nutrient profile, shouldn’t their results find fewer healthy foods than the Mexico study reported in this manuscript?

Answer: Thank you for the relevant comment. We agree, we would expect a higher proportion of products classified as healthy according to the less stringent version used in our study than the one used by Durán et al. However, this inconsistency may be explained by differences in the overall healthiness of products included in both studies. Products in our database were less healthy compared to the ones included in Duran’s study, as suggested by the lower percentage of “healthy” products in our study compared to Duran’s study by the PAHO (38% vs. 20%) or the Chile Phase 3 models (58 vs 33%). We have included this explanation in the discussion section. See paragraph below.

Also, Durán study used Chile’s final phase (i.e., the most stringent nutrient profile) compared with the present study using the Chile’s Phase 1 nutrient profile.

What is the justification for comparing Mexico to Chile Phase 1 rather than Phase 3? If no compelling justification, analyses should be rerun with such comparison.

Answer: Thank you for your comment. We agree, and we have modified the sentence in the discussion and now we are comparing Chilean Phase 3 instead of Chile Phase 1. We did not need to rerun comparisons since these comparisons had already been conducted and shown in supplementary table 4.

“To date, scarce evidence is available regarding calibration of Latin America NP models. In a study conducted in a sample of products from Brazil, Durán et al 2020 [46], classified 38% of the products as healthy according to the PAHO model, while more than half of the products were classified as healthy by Chile Phase 3 (58%) and Brazil (55%) NP models. ~~Similarly,~~ i~~I~~n ~~this~~ our study the PAHO model and the three phases of the Mexican NP model classified the lowest proportion of products as healthy (around 20%), whereas almost half of the products were classified as healthy by the Brazil model, and around a third were classified as healthy by ~~Peru and~~ Chile Phase ~~1~~3 NP model~~s~~, ~~but for Chilean Phase 3 modelthe proportion of products classified as healthy were little more than a third of the sample.~~ Nevertheless, it is important to mention that the profile used by Durán for Brazil was a more stringent version [47] than the recently approved NP used in our study. Hence, one would expect a higher proportion of products classified as healthy according to the less stringent version used in our study than the one used by Durán et al. However, this inconsistency may be explained by differences in the overall healthiness of products included in both studies. Products in our database were less healthy compared to the ones included in Duran’s study, as suggested by the lower percentage of “healthy” products in our study compared to Duran’s study by the PAHO (38% vs. 20%) or the Chile Phase 3 models (58 vs 33%) [46].

# *** Reviewer #3:

I mostly confine my remarks to statistical aspects of this paper. I would mostly change the emphaiss, and I have some issues with the figures.

If I understand correctly, one method of validation was to see what proportion of the total items were called XXX (e.g. unhealthy) in the different NPs. This can't be a very good method of validation. What if the proportions are similar but the foods are different. e.g. if there were only 5 foods (A, B, C, D, E) and one NP labeled A and B as unhealthy while the other labeled D and E, then they would each label 40% but this would be an indication of total lack of validity. So, I would drop that from the write up and just go with kappas across the various foods (I am assuming kappa was calculated for each food, so that you had thousands of 2x2 tables to work with).

Answer: Thank you for the comment. We agree partially with the reviewer, since, as the reviewer suggests, proportions may be similar but the foods may be different. We also agree in saying that the kappa agreement should be considered as the strongest statistical method to evaluate validity, which is already provided for all comparisons in our Supplemental data. Nevertheless, comparing the proportion of healthy and unhealthy products is a common approach used to validate and calibrate nutrient profiles (i. e., doi:10.1017/S0007114518001575, doi:10.1017/S1368980019005056, DOI: 10.1017/S1368980007000377 doi:10.3390/nu10060737). Further, this is the only one analysis that allows comparing the nutrient profiles against each other. The classification of “healthy” or “less healthy” (or “comply” vs “not comply”) foods is one of the steps for testing the construct validity according to Cronbach and Meehl’s (Step 2: Developing scales to measure these theoretical constructs.). This approach is simple and allows to easily compare among nutrient profiles and among validation studies. Therefore, we consider it is important to keep this analytical approach.

I'm not going to comment much on the validity of the whole plan --- I am not a nutritionist --- but on p. 9, line 249, it appears that raw or unprocessed food was not labeled. How can this be right? IN that case, pure butter or olive oil or sugar would not get warnings for being high in calories or fat; sugar would not be high in sugar! I'd think it would be better to distinguish ingredients from finished products. After all, no one makes a meal of just butter or just sugar. Also, earlier the authors talk about dairy products being labeled, does this mean they were only talking about dairy products that are processed? This seems unclear.

Answer: Thank you for the comment. We understand reviewer’s concerns regarding the fact that some ingredients (e.g., olive oil or sugar) may not be considered by nutrient profile models. However, nutrient profile models particularly evaluate the healthiness of a product based on the addition of critical nutrients, such as, sugar, fats or sodium. Culinary ingredients (such as sugar, oil or salt with only 1 ingredient) are not consumed directly from the package, and consumers already know their general nutrition composition, therefore, labeling is not useful for this type of products. Also, raw or unprocessed products have in general better nutrition composition than the similar ones that are processed or ultra-processed (i. e. unprocessed meat vs processed meat, or whole milk vs sugary milk), and in nutrition terms it is more recommended to include unprocessed or raw foods (that will be free of labels) in the diet, than processed or ultraprocessed products (most of them labelled). Therefore, most nutrient profiles do not evaluate raw or unprocessed products, such as the ones mentioned by the reviewer, since they do not have **added** unhealthy ingredients.

In Supplementary table 3 we detail different nutrient profiles, along with their eligibility criteria for processed or ultraprocessed products. For example, they may be defined as those products which are ‘commonly added’ with critical nutrients (i.e. PAHO model, or Peru), or by ‘the addition of critical nutrients’ (i.e. Mexico, Chile, Uruguay). Others, such as the Brazil model, may exclude particular food groups (i.e. fruits, meat, or culinary ingredients). Similarly, the Mexican Phase 3 evaluates the total amount of fat (not only the added fat, as in Mexico’s Phase 1 or 2 nutrient profiles), but only if the product contains added the fat. The same case for sodium or sugar. Some studies compare nutrient profiles among processed or ultraprocessed products (doi:10.3390/nu10060737) while others evaluate all the market share (unprocessed, processed and one ingredient products) because it is important to measure the variability of the products in the market share and how many products will be considered as “healthy” by different nutrient profiles (which include the eligibility criteria of the product that have to be labeled). In supplementary table 1 we describe the packaged foods included in each food group in order to clarify how was or food data base comprised.

Back to statistical issues. For the graphs: None of these are good. Stacked bar plots are not a good graphical method. See the work of William Cleveland. I would suggest Cleveland dot plots. Since you are using Stata, you could use the multidot command

Answer: thank you for the suggestion. We have improved our graphs.
