# Supplementary material for: Evaluation of the Mexican warning label nutrient profile on food products marketed in Mexico in 2016 and 2017: A cross-sectional analysis
Source: PLoS Med. 2022 Apr 20;19(4):e1003968. doi: 10.1371/journal.pmed.1003968 (PMC9067899; doi:10.1371/journal.pmed.1003968)
Supplement: Attachment — Submitted filename: Response to Reviewers_Round2_aj.docx [file pmed.1003968.s012.docx]

**Review for: PLoS Medicine**

Title: Evaluation of the Mexican warning label nutrient profile on food products marketed in Mexico in 2016 and 2017: A cross-sectional analysis.

Manuscript number: PMEDICINE-D-21-02443_R1

June 28, 2021

Due date to resubmit: March 19th, 2022.

# Requests from Editors:

We ask you to adapt the title to better match journal style, and suggest: "Evaluation of the Mexican warning label nutrient profile on food products marketed in Mexico in 2016 and 2017: A cross-sectional analysis".

Answer: Title was adapted as required.

Please remove the hyphen from "Latin America" at line 31 and any other instances.

Answer: Hyphen was removed from line 31.

Please add "... nutrient profiles (NP)" at line 31 and make that "Mexican NP" in the following line.

Answer: NP was added in line 31 and removed Nutrient Profile from the following sentence.

At line 35 we suggest removing "... using the calibration method.", and adding a sentence, say, early in the "Methods and findings" subsection of the abstract to explain briefly what this method involves.

Answer: “using the calibration method” was removed from the background and included in the methods section, where a description of the method was already included.

Please make that "3 implementation phases" at line 39, and use this style throughout the article (although numbers should not be used at the start of sentences).

Answer: we used “3 implementation phases” style throughout the article.

At line 39, do you need to state briefly when the implementation phases were/will be?

Answer: thank you, yes, we included the three years of the implementation phases.

At line 47, please adapt the text to "... were those implemented in Ecuador ..." or similar.

Answer: thank you for the comment, we have adapted the text as suggested.

At line 53, please make that "... are limited"; and "data are ..." at line 562.

Answer: thank you for the correction, we have changed the text as suggested.

At line 53, please rephrase "... as if they were octagon warnings", which may not be clear to all readers (e.g., you could add a few words to explain what an "octagon warning" is. A brief explanation may be needed later in the ms too.

Answer: Thanks for the suggestion. We have edited the text as suggested. We have also included a brief explanation stating that warning octagons are for critical ingredients and warning rectangles for other ingredients. A similar explanation was included in the introduction of the manuscript.

Line 33: “Mexican Nutrient Profile (NP) was developed as part of the new warning label regulation implemented in 2020, considering 5 warning octagons (calories, sugar, sodium, saturated fats, and trans fats) and 2 warning rectangles (caffeine and non-caloric sweeteners).”

Line 135: “). In 2020, Mexico adopted a new mandatory FoPL considering warning ~~labels~~ octagons with the legends (“Excess in…”) ~~[16]~~ for critical ingredients (i.e., calories, sugar, sodium, saturated fats, trans fats~~, non-caloric sweeteners and caffeine)~~ and warning rectangles for products that include caffeine and non-caloric sweeteners, along with the statement “avoid/not recommended in children” [16].”

Line 604: “First, warning ~~legends~~ rectangles (~~such as the addition of~~ caffeine or non-caloric sweeteners) considered by the Mexican NP model were evaluated as if ~~they~~ all of the warnings were octagonal seals ~~warnings~~. Hence, the maximum number ~~of WL~~ for the Mexican NP model was seven, five octagons and two rectangle~~legends~~. A similar approach was used when evaluating Ecuador’s NP, by giving a similar interpretation to both, the amber and the red traffic lights. These decisions may have overestimated the strictness of Mexico and Ecuador NP models. Nevertheless, rectangle~~legends~~ and amber warnings may have equal importance as octagons or red warnings, since they may be used to regulate advertising directed to children, or the use of health and nutrition claims [60].”

At line 53, why do you highlight the Brazil NP when those from three countries appear to have low agreement with the PAHO model? If appropriate, all three countries could be listed here.

Answer: We highlight the Brazil NP because for Chile and Peru they are temporary implementation phases, while Brazil is the final version of the NP. Nevertheless, we included the three countries in the conclusions as suggested.

Line 53: “In contrast, Peru Phase 1, Brazil, and Chile Phase 1 NP models may have limited usefulness…”

Please adapt the author summary so that there are three subsections, each comprising about three points (the third subsection should be titled "What do these findings mean?".

Answer: Thank you for the observation. We have adapted the author summary section as required.

Author summary:

**“What Do These Findings Mean?**

• Our study has some limitations related to the quality of the information reported in the packages of the products included. Nevertheless, ~~O~~our findings add support to the relevance of using the Mexican nutrient profile to evaluate the nutritional quality of food products as a basis for the prevention of obesity and chronic diseases related to diet.”

At line 89 (author summary) please make that "We compared ...".

Answer: we have changed the sentence as required.

Thank you for adding information about the study protocol. PLOS Medicine's aim is for readers to be able to appreciate to what extent a study's analyses were preplanned, or to the contrary could have been data-driven. Therefore, please add a statement in the Methods section (main text) of the type: "This study did not have a prespecified analysis plan"; or "The study's analyses were preplanned, except for ...", for example.

Answer: Thank you for the comment. We have included the statement in the methods section.

“*Latin American Nutrient Profiles*

This study did not have a prespecified analysis plan. According to the most recent version of the NP in Latin America ~~For this study,~~ food and beverages were classified according to different NP from six ~~Latin American~~ countries (Mexico, Chile, Uruguay, Brazil, Peru and Ecuador) considering different stages of implementation, from the first or most permissive to the last or definitive one, as well as the PAHO NP model.”

Please refer to the STROBE checklist in the Methods section (main text), e.g., "The study is reported according to STROBE (see S1_STROBE_Checklist).", and you may wish to add a reference to the primary STROBE publication.

Answer: Thank you for the comment, we have referred the strobe checklist in the manuscript as suggested.

Line 254: “The study is reported according to STROBE ([www.strobe-statement.org](http://www.strobe-statement.org)) (see S5_STROBE_Checklist) [39]. ~~(S5 Strobe checklist)~~”

Does "WL" need to spelt out at line 557?

Answer: Thank you for the observation, we have dropped from the text “of WL” in line 557.

Please remove the information on funding from the end of the main text. In the event of publication, this information will appear in the article metadata, via entries in the submission form.

Answer: thank you for the comment, we have removed funding information from the main text as required.

Noting references 33 & 47, please format the authors' names as for other references.

Answer: Thank you for the correction. We have modified the format for references 33 and 47.

# Comment from Academic Editor:

One minor comment I would suggest would be to include a bit more background in their Introduction and Author summary about the Mexican NP model. As they explained, the PAHO model was created for Latin American countries as reference, however, each country including Mexico have developed their own NP and it's unclear why. Why is the Mexican NP needed then? What is the Mexican NP trying to do that the PAHO is not doing? Both methods are in high agreement and are very similar, so I think the audience would appreciate a bit more information on why the Mexican NP method (and hence its evaluation against PAHO) is needed. Perhaps this should also be briefly mentioned in Discussion.

Answer: We appreciate your comment. We have included in the introduction and author summary the reasons and importance to develop the Mexican NP model.

- In the introduction: “The PAHO model provides regional criteria for acceptable amounts of critical nutrients (salt, sugar, saturated fats and trans fats) in Latin America. However, NP models underpinning FOPL systems should be relevant for the national or regional food supply. Therefore, regional criteria proposed by the PAHO model should be carefully adapted to ensure that the FOPL system is sensitive enough to classify products according to their healthfulness. Classifications produced by the adapted NP models should also align with national dietary guidelines. In Mexico sugar sweetened beverages, particularly cola drinks, are partly responsible for the high prevalence of obesity and diabetes; these drinks are highly consumed by the population, including children. Therefore, adaptations to the PAHO model for its use in Mexico included additional thresholds for calories and a warning for added caffeine, which intake is not recommended among children. Other adaptations included removing the total fat threshold of the PAHO model, since total fat includes healthy fatty acids, which are not common in the Mexican diet and their intake needs to be promoted. The sodium threshold proposed by PAHO was also adapted considering the national market share in Mexico. In accordance to the Mexican FoPL regulation, which considers a 3 implementation phases (2020, 2023 and 2025), 3 NP were proposed, with stricter nutrient criteria for each progressive phase [8,16].”

And then we deleted it from line 186: ~~“In accordance to the Mexican FoPL regulation, which considers a three3-phase implementation phases process (2020, 2023 and 2025), three 3 NP were proposed, with stricter nutrient criteria for each progressive phase [8,16].”~~

In the author summary: “Even when the PAHO model was created for Latin American countries, it is relevant to choose and adapt a NP model to local nutrition policies and epidemiology contexts. Recently, Mexico developed its own nutrient profile model based on the PAHO and Chile Phase 3 models, nutritional scientific population-based recommendations and a feasibility analysis of products sold in the Mexican market share.”

# Comments from Reviewers:

*** Reviewer #1:

Thank you for your efforts.

Answer: thank you for your review which improved the quality of our article.

*** Reviewer #2:

The revised manuscript adequately addressed my concerns from the prior review. I believe it will make a terrific contribution to the literature in global public health and nutrition.

Answer: thank you for your review and comments.

*** Reviewer #3:

One remaining issue is the graphs. I said they were not good, they said they had improved them. But I do not see any changed graphs. Did I miss something? Or did the authors fail to update the figures in the text with new figures? Peter Flom

Answer: Thank you for your comment. In the revised version we have replaced the bars for the Cleveland dot plots only for Graph 1 since most of the articles similar to this use Stacked bars (doi:10.1017/S0007114518001575,) or simple bars (doi:10.1017/S1368980019005056, doi: 10.1017/S1368980015002748, DOI: 10.1017/S1368980007000377). The remaining graphs were not replaced since we believe that the suggested graphs do not allow the reader to easily capture the distribution of products across the number of warnings. (see below)

Graph 2A

Graph 2B

Suppl. Graph 1
